# Localized Semaglutide Injection for Hyperinsulinemia-Induced Lymphatic Dysfunction: A Narrative Review Proposing a Promising Metabolic Perspective for Lymphedema Therapy

**DOI:** 10.34172/apb.025.43911

**Published:** 2025-09-04

**Authors:** Maher Monir. Akl, Amr Ahmed

**Affiliations:** ^1^Faculty of Medicine, Novosibirsk State University, Novosibirsk, Russia; ^2^The Public Health Department, Riyadh First Health Cluster, Ministry of Health, Riyadh, Saudi Arabia

**Keywords:** Lymphedema, Insulin resistance, GLP-1 receptor agonists, Lymphangiogenesis, Metabolic dysfunction, Vascular inflammation

## Abstract

Lymphedema, traditionally viewed as a mechanical consequence of lymphatic obstruction, is increasingly recognized as a complex disorder rooted in metabolic dysfunction, particularly insulin resistance and chronic hyperinsulinemia. This paradigm-shifting hypothesis redefines lymphedema as a vascular complication driven by systemic metabolic stress, where prolonged hyperinsulinemia impairs lymphatic endothelial cell (LEC) function, triggering inflammation, oxidative stress, and structural damage. Insulin resistance disrupts the phosphoinositide 3-kinase (PI3K)/AKT signaling pathway, critical for lymphangiogenesis and endothelial integrity, leading to compromised lymphatic drainage. Pro-inflammatory cytokines, such as tumor necrosis factor-alpha (TNF-α) and interleukin-6 (IL-6), exacerbate this dysfunction by activating nuclear factor kappa-light-chain-enhancer of activated B cells (NF-κB) and promoting reactive oxygen species (ROS) production, while advanced glycation end products (AGEs) engaging RAGE amplify fibrosis and endothelial apoptosis. Glucagon-like peptide-1 receptor agonists (GLP-1RAs), such as liraglutide and semaglutide, offer a revolutionary therapeutic approach by addressing both metabolic and vascular components of lymphedema. By enhancing PI3K/AKT signaling, GLP-1RAs restore insulin sensitivity, mitigate hyperinsulinemia, and suppress inflammatory pathways (NF-κB, TLR4). Their activation of VEGF-C/VEGFR-3 and endothelial nitric oxide synthase (eNOS)/NO pathways promotes lymphangiogenesis and reduces ROS-induced damage, enhancing lymphatic vessel repair. Clinical evidence, including a 2024 case report, demonstrates significant reductions in limb volume (from 10.3% to 3.4%) and restored lymphatic function in breast cancer-related lymphedema following GLP-1RA therapy. Localized administration optimizes therapeutic outcomes by targeting LECs, minimizing systemic side effects. This narrative review synthesizes lymphedema’s metabolic pathophysiology, proposes localized semaglutide as a novel therapy, and suggests experimental protocols to advance lymphedema management.

## Introduction

 Lymphedema is a chronic, progressive disorder characterized by the accumulation of protein-rich lymphatic fluid in interstitial spaces, primarily affecting the limbs but potentially involving other body regions. This condition results from impaired lymphatic drainage due to dysfunctional lymphatic vessels, leading to tissue edema, chronic inflammation, and progressive fibrosis.^1^ Over time, lymphedema induces significant structural and functional changes in affected tissues, driven by cellular alterations, including proliferation of fibroblasts, adipocytes, and immune cells, notably macrophages.^2^ The cytobiological environment of lymphedema is closely tied to chronic low-grade inflammation, mediated by pro-inflammatory cytokines such as tumor necrosis factor-alpha (TNF-α), interleukin-6 (IL-6), and interleukin-1β (IL-1β).^3^ These cytokines promote extracellular matrix (ECM) remodeling and fibrosis, further impairing lymphatic function and perpetuating tissue damage.^4^ A key feature of lymphedema is localized adipogenesis, where excessive adipose tissue accumulation exacerbates fluid retention and lymphatic dysfunction. This process is particularly pronounced in obesity, where adipose tissue, as an endocrine organ, secretes pro-inflammatory adipokines, including leptin and resistin, which impair lymphatic function by enhancing inflammation and disrupting vascular homeostasis.^5,6^ Additionally, the mechanical burden of excess adipose tissue obstructs lymphatic flow, compromising vessel architecture and amplifying systemic inflammation, thus accelerating the progression of lymphedema and obesity.^7^ Insulin resistance, a hallmark of obesity and metabolic dysfunction, plays a pivotal role in this pathological cycle. It reduces the responsiveness of tissues such as skeletal muscle, liver, and adipose tissue to insulin, leading to compensatory hyperinsulinemia. This chronic hyperinsulinemic state induces endothelial dysfunction in lymphatic endothelial cells (LECs), impairing lymphatic drainage and exacerbating tissue inflammation.^8^ Hyperinsulinemia also activates the nuclear factor kappa-light-chain-enhancer of activated B cells (NF-κB) pathway, increasing oxidative stress and promoting fibrosis in lymphatic vessels.^9^ Furthermore, dysregulated adipokine secretion in obesity marked by elevated leptin and resistin and reduced adiponectin levels disrupts insulin signaling, perpetuating lymphatic dysfunction through sustained inflammation and ECM remodeling.^10^ Elevated free fatty acids (FFAs) in obesity further contribute to lipotoxicity, compounding damage to both metabolic and lymphatic systems.^11^

 In this review, we investigate the interplay between insulin resistance and lymphatic vessel dysfunction as a novel mechanism underlying lymphedema progression. We also explore a therapeutic approach utilizing local injections of Glucagon-like peptide-1 (GLP-1) receptor agonists to improve insulin sensitivity and modulate lymphatic endothelial function, offering a potential strategy to address the metabolic and vascular components of this debilitating condition.

## Methodology

 This narrative review, combined with a hypothesis proposing a metabolic perspective for lymphedema therapy, investigates lymphedema as a condition influenced by metabolic dysfunction, particularly insulin resistance and chronic hyperinsulinemia, and evaluates localized glucagon-like peptide-1 receptor agonists, such as semaglutide, as a potential therapeutic approach.

###  Evidence synthesis

 A systematic literature synthesis was conducted to integrate findings from immunometabolism, vascular biology, redox signaling, and lymphatic pathophysiology. We searched PubMed, Scopus, Web of Science, and Google Scholar using Medical Subject Headings and free-text terms, including “lymphedema,” “insulin resistance,” “hyperinsulinemia,” “lymphatic endothelial cells,” “lymphangiogenesis,” “glucagon-like peptide-1 receptor agonists,” “phosphoinositide 3-kinase/AKT,” “nuclear factor kappa-light-chain-enhancer of activated B cells,” “Toll-like receptor 4,” “reactive oxygen species,” and “advanced glycation end products.” Boolean operators ensured comprehensive coverage of peer-reviewed studies from 2000 to 2025, capturing foundational and recent insights into metabolic-vascular interactions. From 1,456 articles, 312 duplicates were removed, leaving 1,144 for title and abstract screening. Of these, 789 were excluded due to irrelevance, lack of mechanistic focus, non-English language, or inaccessible full texts. Full-text evaluation of 355 articles, based on inclusion criteria (relevance to insulin signaling, lymphatic dysfunction, glucagon-like peptide-1 receptor agonist mechanisms, and clinical outcomes), yielded 56 studies. These included in vitro studies of LEC function, animal models of insulin resistance, human biomarker data on inflammation (TNF-α, IL-6) and oxidative stress (reactive oxygen species, advanced glycation end products), and a 2024 case report showing limb volume reduction with glucagon-like peptide-1 receptor agonists. While this single case provides preliminary evidence, it is insufficient to establish efficacy, highlighting the critical need for controlled clinical trials to validate the therapeutic potential of glucagon-like peptide-1 receptor agonists in lymphedema management. The synthesis, adhering to the SANRA framework (scoring 11/12 for clarity and evidence integration), developed a mechanistic model linking insulin resistance-induced disruptions in phosphoinositide 3-kinase/AKT and vascular endothelial growth factor-C/VEGFR-3 signaling, amplified by nuclear factor kappa-light-chain-enhancer of activated B cells, Toll-like receptor 4, and receptor for advanced glycation end products-mediated inflammation, to lymphatic dysfunction.

###  Proposed experimental directions

 To advance this hypothesis, we suggest experimental protocols distinct from the evidence synthesis: proteomic profiling to assess insulin signaling defects in LECs; metabolomic analysis of inflammatory and oxidative stress markers in lymphatic tissues; in vitro studies of LEC function under hyperinsulinemic conditions; animal models to evaluate localized glucagon-like peptide-1 receptor agonist injections on lymphatic repair and glucose homeostasis; and clinical trials to assess localized semaglutide therapy, monitored via lymphoscintigraphy or magnetic resonance imaging.

## Biochemical pathways linking obesity-induced inflammation to insulin resistance: The role of pro-inflammatory cytokines TNF-α and IL-6

 Obesity drives a chronic state of low-grade inflammation that significantly contributes to insulin resistance, primarily through adipose tissue expansion and immune cell infiltration, particularly pro-inflammatory M1 macrophages.^12^ These macrophages secrete key pro-inflammatory cytokines, including TNF-α and IL-6, which disrupt insulin receptor signaling and impair glucose homeostasis.^13^ TNF-α plays a central role in insulin resistance by activating serine kinases, such as c-Jun N-terminal kinase (JNK) and IκB kinase (IKK).^14^ These kinases phosphorylate insulin receptor substrate-1 (IRS-1) at serine residues, impairing its ability to propagate insulin signals via the phosphoinositide 3-kinase (PI3K)-AKT pathway, which is critical for glucose uptake in skeletal muscle and adipocytes.^15,16^ Additionally, TNF-α downregulates glucose transporter type 4 (GLUT4) expression, further compromising glucose transport and exacerbating insulin resistance.^17^

 IL-6 contributes to insulin resistance through a complementary mechanism by activating the signal transducer and activator of transcription 3 (STAT3) pathway, which induces suppressor of cytokine signaling-3 (SOCS-3) expression. SOCS-3 inhibits insulin receptor activity and IRS-1 function, disrupting PI3K-AKT signaling.^18^ Moreover, IL-6 promotes hepatic gluconeogenesis and dysregulates lipid metabolism, leading to ectopic fat deposition in the liver, a key contributor to systemic insulin resistance.^19^ Excess FFAs, a hallmark of obesity, exacerbate insulin resistance and lymphatic vascular dysfunction through multiple mechanisms, including lipotoxicity, mitochondrial dysfunction, and oxidative stress.^20^ In non-adipose tissues, FFAs accumulate as diacylglycerols (DAGs), activating protein kinase C (PKC), which phosphorylates IRS-1 at serine/threonine residues, inhibiting PI3K-AKT signaling and impairing glucose uptake.^21^ Ceramide, another lipid intermediate, accumulates during lipotoxicity, promoting apoptosis and further disrupting IRS-1 function, contributing to β-cell dysfunction and systemic metabolic dysregulation.^22^ Excess FFAs also overload mitochondria, leading to incomplete fatty acid oxidation, accumulation of toxic intermediates, and reduced ATP production, particularly in skeletal muscle, a critical tissue for insulin-mediated glucose uptake.^23^

 This mitochondrial overload generates reactive oxygen species (ROS), causing oxidative stress that damages mitochondrial DNA, proteins, and membranes, further impairing bioenergetics and exacerbating insulin resistance. ROS also activates stress-related kinases (JNK and IKK), which phosphorylate IRS-1 and enhance secretion of TNF-α and IL-6, perpetuating chronic inflammation in obese adipose tissue.^24^ Additionally, FFAs induce endoplasmic reticulum (ER) stress, disrupting protein folding and triggering the unfolded protein response (UPR), which activates JNK and increases ROS production, further worsening insulin resistance. These cascades have profound implications for lymphatic vascular health. Oxidative stress and excess FFAs impair LEC function, disrupting mitochondrial bioenergetics and promoting lipid peroxidation, which increases vascular permeability and reduces lymphatic drainage, contributing to lymphedema.^24^ Furthermore, the interplay between expanding adipose tissue and the lymphatic system creates a vicious cycle, where inflammation driven by pro-inflammatory macrophages impairs lymphatic function, exacerbating fluid retention and tissue damage.^25^

## The interrelationship between insulin resistance and lymphatic dysfunction: A detailed exploration of molecular mechanisms and pathophysiological consequences

 Insulin resistance, characterized by reduced cellular responsiveness to insulin in tissues such as skeletal muscle, adipose tissue, and liver, disrupts metabolic homeostasis and significantly impacts the lymphatic vasculature. This condition triggers compensatory hyperinsulinemia, where pancreatic β-cells secrete excessive insulin to maintain glucose homeostasis, contributing to endothelial dysfunction, chronic inflammation, and structural damage to lymphatic vessels.^26^ The molecular basis of insulin resistance involves disruptions in insulin signaling pathways, with widespread metabolic and vascular consequences.

 Under physiological conditions, insulin binds its receptor, activating IRS-1, which propagates signals through the PI3K/AKT pathway, essential for glucose uptake via GLUT4 translocation to the cell membrane.^27^ In insulin resistance, pro-inflammatory cytokines, such as TNF-α and IL-6, promote serine phosphorylation of IRS-1, impairing its interaction with PI3K and disrupting AKT signaling, leading to reduced glucose uptake and exacerbated hyperglycemia.^28,29^ Chronic hyperinsulinemia aggravates LEC dysfunction, critical for lymphatic vessel integrity. Normally, insulin supports lymphangiogenesis and LEC proliferation via PI3K/AKT and extracellular signal-regulated kinase (ERK) pathways.^30,31^ However, in insulin resistance, dysregulated insulin signaling impairs these processes, reducing lymphangiogenesis, lymphatic drainage, and increasing inflammation in lymphatic vessels.

 This is exacerbated by PKC activation and ROS generation, which drive endothelial damage and activate nuclear factor kappa-light-chain-enhancer of activated B cells (NF-κB), a key regulator of inflammatory responses, perpetuating a feedback loop that worsens insulin resistance and systemic inflammation.^32^

 Adipose tissue dysfunction, a hallmark of obesity and a driver of insulin resistance, further impairs lymphatic function. Hypertrophic adipose tissue releases pro-inflammatory cytokines and FFAs, which activate Toll-like receptor 4 (TLR4) on LECs, inducing cytokine and chemokine production, lymphatic vessel leakage, fibrosis, and reduced fluid clearance.^33^

 Advanced glycation end products (AGEs), which accumulate in insulin-resistant states due to chronic hyperglycemia, exacerbate lymphatic dysfunction. AGEs crosslink with ECM components in lymphatic vessels, engaging the receptor for AGEs (RAGE) and activating NF-κB, which triggers pro-inflammatory mediator release, endothelial cell apoptosis, and fibrosis.^34,35^ This RAGE-NF-κB axis impairs lymphatic function and contributes to systemic insulin resistance by disrupting interstitial fluid balance and immune cell trafficking.^36^ The interplay between insulin resistance and lymphatic dysfunction involves a complex network of pro-inflammatory cytokines, oxidative stress, adipose tissue dysfunction, and AGE accumulation. Disrupted insulin signaling directly impairs LEC function, while chronic inflammation and oxidative stress exacerbate lymphatic vessel damage. These interconnected pathways highlight the need for therapeutic strategies targeting both glucose metabolism and the inflammatory and vascular complications of insulin resistance to improve metabolic and lymphatic health.^37^

## Exploring the therapeutic approach of local GLP-1 agonist injections as a promising treatment for lymphedema

 GLP-1 receptor agonists, such as semaglutide and liraglutide, have emerged as promising therapeutic agents for managing metabolic and vascular dysfunctions, including lymphedema, a condition exacerbated by insulin resistance. GLP-1 receptors, expressed on pancreatic β-cells, vascular endothelium, and LECs, mediate dual effects that enhance insulin sensitivity and protect vascular integrity, offering a novel approach to treating lymphatic disorders.^38^ At the molecular level, GLP-1 receptor agonists enhance glucose-stimulated insulin secretion and promote β-cell survival by activating cyclic AMP (cAMP)-dependent signaling. This cascade phosphorylates protein kinase A (PKA), activating transcription factors such as cAMP response element-binding protein (CREB), which supports insulin production and prevents β-cell apoptosis.^39^

 Additionally, GLP-1 agonists improve insulin sensitivity in peripheral tissues by enhancing glucose uptake via the PI3K/AKT pathway,^40^ mitigating hyperglycemia and reducing compensatory hyperinsulinemia, a key contributor to lymphatic endothelial dysfunction.^41^

 From a vascular perspective, GLP-1 receptor agonists exhibit vasoprotective effects that counteract lymphatic endothelial damage. Activation of GLP-1 receptors on LECs stimulates endothelial nitric oxide synthase (eNOS), increasing nitric oxide (NO) production, which promotes vasodilation and reduces oxidative stress.^42^ This is critical in insulin-resistant states, where elevated ROS and inflammation impair endothelial function. GLP-1 agonists suppress ROS production,^43^ and inhibit pro-inflammatory pathways, notably NF-κB,^44^ preserving lymphatic vessel integrity and supporting lymphangiogenesis, essential for effective lymphatic drainage. In obese and insulin-resistant individuals, adipose tissue dysfunction drives lymphatic impairment through the release of pro-inflammatory cytokines and FFAs.^45^

 GLP-1 receptor agonists mitigate this by suppressing inflammatory mediator release and downregulating TLR4 expression on LECs, which is typically upregulated by excess FFAs.^46^ Reduced TLR4 signaling attenuates lymphatic inflammation, preventing vessel leakage, fibrosis, and impaired fluid clearance.^47^ Furthermore, GLP-1 agonists decrease the accumulation of AGEs in vascular tissues,^48^ which otherwise crosslink ECM proteins and activate the RAGE, perpetuating NF-κB-driven inflammation and endothelial dysfunction.^49^ Additionally, GLP-1 agonists reduce the expression of pro-fibrotic markers, such as transforming growth factor-beta (TGF-β),^50^ which is elevated in lymphatic dysfunction and contributes to fibrosis.^51^ By targeting both metabolic and vascular components, local GLP-1 receptor agonist injections offer a promising therapeutic strategy for lymphedema, addressing insulin resistance, inflammation, and lymphatic dysfunction through interconnected molecular pathways.

## Promising clinical evidence

 Recent clinical evidence supports the therapeutic potential of glucagon-like peptide-1 receptor agonists (GLP-1RAs) in managing breast cancer-related lymphedema. A 2024 case report described significant symptom resolution in a patient with severe lymphedema following breast cancer surgery and adjuvant therapy, after initiating GLP-1RA therapy for weight loss. The patient exhibited a reduction in limb volume from 10.3% to 3.4% after 13 months of treatment, alongside a 24% body weight reduction and restored lymphatic pumping function, confirmed by imaging. These improvements enhanced the patient’s quality of life, eliminating the need for compression garments.^52^

 These findings suggest that GLP-1RAs address the pathological interplay between insulin resistance and lymphatic dysfunction. Insulin resistance impairs LEC function through chronic inflammation, oxidative stress, and disrupted insulin signaling pathways, including PI3K/AKT. By improving insulin sensitivity, GLP-1RAs reduce hyperinsulinemia, a key driver of lymphatic vessel dysfunction.^52^ Mechanistically, GLP-1RAs activate GLP-1 receptors on LECs, promoting lymphangiogenesis and lymphatic vessel repair via nitric oxide (NO) production through eNOS and suppression of ROS.^53^ Additionally, GLP-1RAs downregulate pro-inflammatory cytokines and TLR signaling, particularly TLR4, reducing lymphatic inflammation and preventing fibrosis.^54^

 This case underscores the dual benefits of GLP-1RAs in improving metabolic and lymphatic health, potentially reducing reliance on invasive interventions like lymphovenous bypass or vascularized lymph node transplantation. Further studies are needed to elucidate the precise mechanisms by which GLP-1RAs enhance LEC function and to validate their efficacy in larger cohorts with secondary lymphedema.^52^

## Localized administration of GLP-1 receptor agonists: A promising therapeutic strategy for lymphedema

 Localized administration of GLP-1RAs, such as liraglutide or semaglutide, offers a promising therapeutic strategy for secondary lymphedema, particularly post-breast cancer surgery. This approach targets both metabolic disturbances and lymphatic dysfunction by restoring insulin signaling, reducing inflammation, and promoting lymphangiogenesis. GLP-1RAs enhance endothelial cell function by inhibiting ROS production and activating NO pathways via eNOS, thereby improving lymphatic drainage and reducing vascular permeability.^53,54^ We propose a localized administration protocol starting with a weekly subcutaneous injection of 0.6 mg liraglutide or 0.25 mg semaglutide into the affected lymphatic region. This targeted delivery ensures high local drug concentrations while minimizing systemic side effects. Over 12 weeks, dosages may be titrated to 1.8 mg for liraglutide or 1 mg for semaglutide, based on clinical response, such as reduced limb volume and improved lymphatic function.^55^ By directly activating GLP-1 receptors on LECs, this approach stimulates lymphatic vessel repair and enhances lymphatic pumping, addressing the core pathology of lymphedema.^55^ Combining this treatment with conventional therapies, such as compression and physical exercise, can optimize outcomes. Monitoring via imaging techniques, such as lymphoscintigraphy or MRI, can assess lymphatic repair and drainage efficacy, providing insights into treatment progress.^56^

## Discussion

 The hypothesis that chronic hyperinsulinemia, driven by insulin resistance, plays a central role in lymphedema pathogenesis represents a transformative shift, redefining this condition as a metabolic and vascular disorder rather than solely a mechanical consequence of lymphatic obstruction. Traditionally, lymphedema has been attributed to physical damage from cancer-related surgeries or radiotherapy. However, emerging evidence positions insulin resistance as a key driver of LEC dysfunction, promoting inflammation, structural damage, and impaired lymphatic drainage, thus offering a novel perspective on its etiology. At the molecular level, insulin resistance disrupts the PI3K/AKT signaling pathway, critical for lymphangiogenesis, glucose uptake, and endothelial function. Under normal conditions, this pathway supports lymphatic vessel repair and fluid homeostasis. In insulin-resistant states, pro-inflammatory cytokines, such as TNF-α and IL-6, induce serine phosphorylation of IRS-1, inhibiting PI3K/AKT signaling and exacerbating hyperglycemia.^28^ This leads to oxidative stress via ROS and PKC activation, compromising LEC integrity, increasing vascular permeability, and promoting inflammation hallmarks of lymphedema.^32^ Additionally, insulin resistance accelerates the formation of AGEs, which engage the RAGE, activating NF-κB. This cascade triggers endothelial cell apoptosis, fibrosis, and chronic inflammation, further impairing lymphatic fluid clearance.^34,35^

 The therapeutic potential of GLP-1RAs offers a revolutionary approach to address this metabolic-driven pathophysiology. GLP-1RAs, such as liraglutide and semaglutide, restore insulin sensitivity by enhancing PI3K/AKT signaling, reducing hyperinsulinemia, and mitigating inflammation through suppression of TNF-α, IL-6, and TLR4 activity.^44,46^ By activating GLP-1 receptors on LECs, these agents stimulate lymphangiogenesis via vascular endothelial growth factor-C (VEGF-C)/VEGFR-3 signaling and enhance endothelial function through eNOS-mediated NO production, counteracting ROS-induced damage.^42,53^ Clinical evidence, including a 2024 case report, demonstrates significant reductions in limb volume and restored lymphatic function in lymphedema patients treated with GLP-1RAs, particularly those with metabolic disorders.^52^ These findings highlight the dual benefits of GLP-1RAs in addressing insulin resistance and promoting lymphatic repair, potentially reducing reliance on invasive interventions like lymphovenous bypass.

 The localized administration of GLP-1RAs represents a groundbreaking strategy, enabling targeted delivery to affected lymphatic regions. This approach maximizes drug efficacy, enhances VEGF-C/VEGFR-3-driven lymphangiogenesis, and minimizes systemic side effects, offering a precise intervention for lymphatic vessel repair.^55^ By integrating metabolic and vascular repair mechanisms, GLP-1RAs could redefine lymphedema management, transforming it from a symptom-driven approach to one that targets its metabolic roots, paving the way for personalized and effective therapies.

## Conclusion

 This review proposes a novel perspective that redefines lymphedema as a metabolic and vascular disorder driven by insulin resistance and chronic hyperinsulinemia. By linking lymphatic dysfunction to systemic metabolic stress, this model emphasizes the interplay between disrupted insulin signaling, chronic inflammation, and oxidative stress, which impair LEC function and fluid homeostasis. Glucagon-like peptide-1 receptor agonists emerge as a promising therapeutic strategy, addressing both metabolic and vascular components. These agents enhance insulin sensitivity, reduce inflammation, and promote lymphangiogenesis, offering a multifaceted approach to restore lymphatic function. Localized administration further optimizes efficacy by targeting affected tissues, potentially transforming lymphedema management from palliative to curative. Future research should focus on clinical trials to validate these findings and standardize protocols, paving the way for innovative therapies that address the metabolic roots of lymphedema, ultimately improving patient outcomes and quality of life worldwide.

## Competing Interests

 The authors declare that there are no conflicts of interest.

## Ethical Approval

 Not applicable.
